# Posttranslational Modifications of Lipid-Activated Nuclear Receptors: Focus on Metabolism

**DOI:** 10.1210/en.2016-1577

**Published:** 2016-12-07

**Authors:** Natalia Becares, Matthew C. Gage, Inés Pineda-Torra

**Affiliations:** Centre for Clinical Pharmacology, Division of Medicine, University College of London, London WC1E 6JF, United Kingdom

## Abstract

Posttranslational modifications (PTMs) occur to nearly all proteins, are catalyzed by specific enzymes, and are subjected to tight regulation. They have been shown to be a powerful means by which the function of proteins can be modified, resulting in diverse effects. Technological advances such as the increased sensitivity of mass spectrometry–based techniques and availability of mutant animal models have enhanced our understanding of the complexities of their regulation and the effect they have on protein function. However, the role that PTMs have in a pathological context still remains unknown for the most part. PTMs enable the modulation of nuclear receptor function in a rapid and reversible manner in response to varied stimuli, thereby dramatically altering their activity in some cases. This review focuses on acetylation, phosphorylation, SUMOylation, and *O*-GlcNAcylation, which are the 4 most studied PTMs affecting lipid-regulated nuclear receptor biology, as well as on the implications of such modifications on metabolic pathways under homeostatic and pathological situations. Moreover, we review recent studies on the modulation of PTMs as therapeutic targets for metabolic diseases.

## Teaser

**Precis:** “Post-translational modifications affect lipid-regulated nuclear receptor biology. This review focuses on how modified FXR, LRH-1, LXR and RXR receptors affect metabolic pathways in homeostatic and pathological contexts.”

Posttranslational modifications (PTMs), or the covalent modification of a protein catalyzed by enzymes, increase the functional diversity of proteins. To aid the scientific community in keeping updated with the number and abundance of modifications, resources such as PTMCuration have been created (1, 2). More than 200 different PTMs have been described so far for both prokaryotic and eukaryotic cells, including ubiquitination, phosphorylation, glycosylation, acetylation, and methylation (3, 4). The discovery of new methodologies and tools to identify PTMs, for example, antibodies specifically recognizing modified residues, proximity ligation assays (5), and the refinement of mass spectrometry–based techniques (6, 7), have allowed us to more easily further our understanding of how these processes are regulated endogenously and how they impact protein–protein interactions. Moreover, gene targeting approaches have also facilitated the study of their impact on mammalian physiology (8).

Nuclear receptors (NRs) are sequence-specific transcription factors primarily regulated through ligand binding. PTMs allow for NR modulation in a fast, reversible manner, inducing specific molecular changes in response to several stimuli that further regulate the receptor’s activity. It has become evident that complex relationships exist between different types of PTMs, which may function in a cooperative or competitive manner (9, 10). This review focuses on the 4 most studied PTMs affecting lipid-regulated NR biology excluding the peroxisome proliferator–activated receptors (PPARs), which have been extensively reviewed elsewhere (11–14).

## Posttranslational modifications

Acetylation of lysine residues was initially identified in histones for their critical role in the control of gene expression (15). Enzymes that add or remove acetyl groups from proteins are named histone acetyltransferases and histone deacetylases (HDACs), respectively. Approximately 85% of all eukaryotic nonhistone proteins are acetylated (16). In mammals, there are 2 different families of HDACs, that is, the classical HDAC family (comprising HDACs 1 to 10) and the sirtuin family of nicotinamide adenine dinucleotide^+^–dependent deacetylases, also known as type III HDACs (16). HDACs can act as part of large multiprotein complexes. For example, HDAC1 and HDAC2 are present in the Sin3 complex, where they interact with the NR corepressor (NCoR) (17) and silencing mediator of retinoic acid and thyroid hormone receptor (18) corepressors. HDAC3 also binds these corepressors, albeit within distinct complexes (19, 20).

Emerging evidence shows that the acetylated state of nuclear proteins, such as NRs including the farnesoid X receptor (FXR) and the liver X receptor (LXR), is regulated in response to several metabolites and cofactors, including nicotinamide adenine dinucleotide^+^ and acetyl coenzyme A (21). Indeed, protein acetylation is altered in obese individuals (22, 23), supporting the link between dysregulation of PTMs and metabolic disease.

GlcNAcylation is the addition and removal of a single sugar modification, *O*-linked *β*-*N*-acetylglucosamine (GlcNAc), to the hydroxyl groups of serine and/or threonine residues of target proteins. Most of this modification is found on intracellular proteins, and about a fourth of all identified *O*-GlcNAcylated proteins are involved in transcription or translation (24). GlcNAcylation is catalyzed by uridine diphospho-*N*-acetylglucosamine:polypeptide *β*-*N*-acetylglucosaminyltransferase and removed by *O*-GlcNAcase in response to several energetic and nutritional stimuli, including glucose (25) and phosphatidylinositol-3,4,5-trisphosphate (26), a mediator in the insulin signaling pathway. The activity of these enzymes is strictly regulated and several pathologies have been linked to aberrant GlcNAcylation, including Alzheimer’s disease and insulin resistance (24).

Phosphorylation is defined as the covalent modification of phosphate groups to specific amino acids, with the most common in eukaryotic cells being serine, threonine, and tyrosine. Phosphorylation is catalyzed by kinases, and removal of phosphate groups is performed by phosphatases. These processes regulate almost every basic cellular process (27). Phosphorylation of NRs can alter protein–protein interactions, protein conformation, and binding of the receptor to DNA, thus affecting their transcriptional activity (28). The complex crosstalk between protein phosphorylation and metabolism has been reviewed recently (29).

Both *O*-GlcNAcylation and phosphorylation occur at serine and threonine residues and thus can compete for the same or adjacent sites within the same protein. This can occur in a reciprocal manner or synergistically, resulting in a complex interplay that may lead to multiple regulatory scenarios (30, 31).

SUMOylation is the covalent binding or conjugation of members of the small ubiquitin-like modifier (SUMO) family to proteins. In mammals, the SUMO family consists of 3 members: SUMO-1, SUMO-2, and SUMO-3 (32). SUMOylation is reversible and uses a specific set of enzymes for processing and attachment—such as the E1 SUMO-activating enzyme subunits 1/2 or members of the E3 ligases protein inhibitor of activated signal transducer and activator of transcription (PIAS) family (33)—and removal, known as SUMO peptidases. In mammals, the enzymes responsible for SUMO removal are referred to as sentrin-specific proteases, deSUMOylating isopeptidases 1 and 2 (34), as well as ubiquitin-specific protease–like 1 (35). How intracellular metabolism regulates protein SUMOylation requires further study.

## Posttranslational Modifications of NRs

### FXR

FXR regulates the expression of numerous genes in response to certain bile acids to regulate bile acid, lipid, and glucose homeostasis (36). Consistent with its metabolic role, this receptor is highly expressed in the liver and small intestine. The modulation of FXR activity is currently being studied for the treatment of metabolic diseases such as dyslipidemia, insulin resistance (37, 38), and nonalcoholic fatty liver disease (39).

#### FXR acetylation

**Figure 1.**
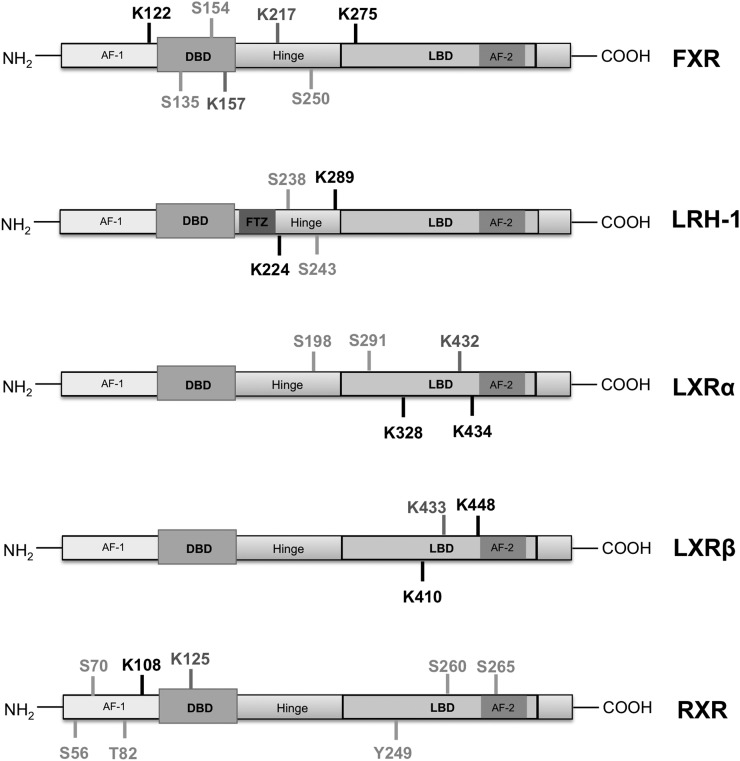
Posttranslational modifications on lipid-activated NRs. Residues modified by acetylation (dark gray), phosphorylation (light gray), and SUMOylation (black) are shown.

**Figure 2.**
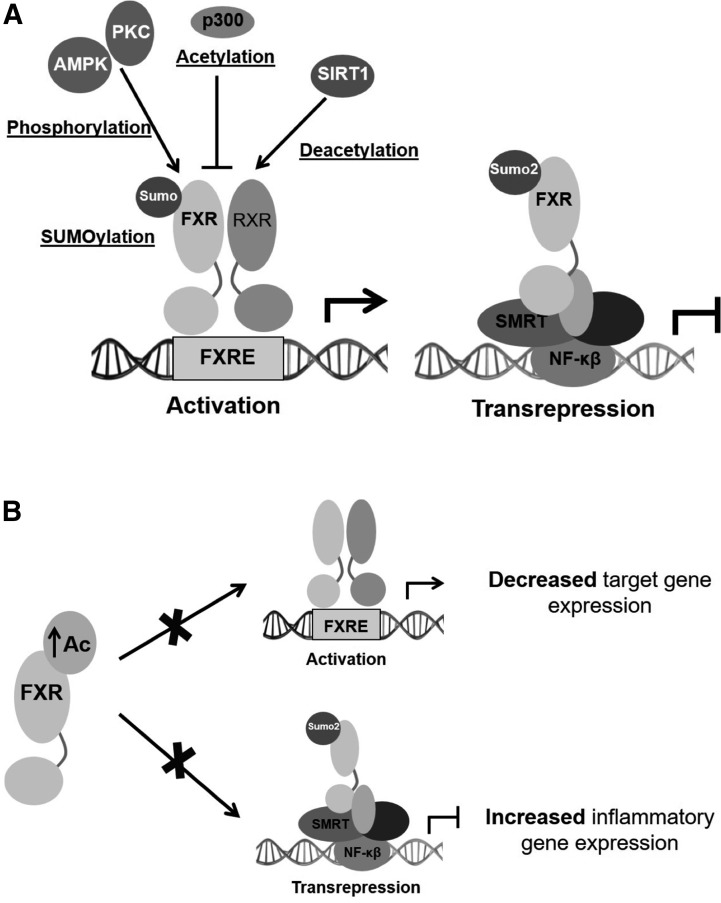
Changes in posttranslational modifications and their effects on FXR activity under physiological (A) or pathological (B) conditions. In homeostatic conditions (A), there is a tight regulation between p300-mediated acetylation and Sirt1-mediated deacetylation of FXR. In parallel, other modifications such as phosphorylation and SUMOylation have also been proven to regulate FXR actions. However, a pathological increase in FXR acetylation (B) and subsequent dysregulated FXR activity leads to increased inflammatory gene expression and decreased target gene expression.

p300 regulates FXR transactivation through acetylation of histones at the promoters of some of its target genes and of the receptor itself [Figs. 1 and 2(A)] (40). The acetylase activity of p300 is increased by FXR agonists, and its inhibition significantly reduces the expression of small heterodimer partner, a well-established FXR target gene that also regulates bile acid synthesis enzymes. Recently, contrasting data have shown that FXR acetylation at lysine 217 and lysine 157 increases protein stabilization but decreases FXR heterodimerization with retinoid X receptor (RXR)*α* and DNA binding, reducing in turn its transactivation capacity (Fig. 1; Table 1) (23). In this way, the p300 acetyltransferase plays a dual role by first initiating FXR target gene expression by acetylating H3 histones, followed by limiting FXR activity by inducing the receptor’s acetylation, which weakens its association with DNA. This process is reciprocally regulated by the sirtuin 1 (SIRT1) deacetylase. Downregulation of endogenously expressed SIRT1 in mouse liver increases acetylation of FXR [Fig. 2(A)]. Conversely, activation of SIRT1 by resveratrol reduces acetylation of FXR in obese mice, leading to an improved metabolic profile. In this model, FXR activity is tightly regulated by the opposing actions of p300 and SIRT1, and this dynamic mechanism has been proposed to be dysregulated in metabolic diseases (Table 1). However, the metabolic outcome seen on the SIRT1-deficient mice could also be explained by effects on other NRs targeted by this deacetylase, including the LXRs (50). Investigations using FXR mutant knock-in models with altered lysine acetylation continue to help us understand the impact of FXR acetylation on metabolic diseases such as obesity (41).

**Table 1.** **Summary of NR Posttranslational Modifications to Date**

Nuclear Receptor	Modification	Residue	Mechanism	Effect on Activity	*In Vivo* Effects	References
FXR	Acetylation	Lys157	Increase protein stabilization	↓	Present in livers of obese mice, increases hepatic inflammation	Fang *et al*., 2008 (40); Kemper *et al*., 2009 (23); Kim *et al*., 2015 (41)
		Lys217	Decreased heterodimerization and DNA binding			

	Phosphorylation	Ser135	Increased binding to coactivator	↑	Decrease leads to bile acid accumulation and hepatic failure	Gineste *et al*., 2008 (42)
		Ser154	Nuclear localization			
		Ser250	Decreased binding to coactivator	↓	Induction leads to bile acid accumulation and hepatic injury	Lien *et al*., 2014 (43)
	SUMOylation	Lys122 (*SUMO1*)	Decreased recruitment to gene promoters	↓ Transactivation	Ameliorates hepatic inflammation and improves metabolic phenotype in obese mice with hyperacetylated FXR	Balasubramaniyan *et al.*, 2013 (44); Kim *et al*., 2015 (41)
		Lys275 (*SUMO1* and *SUMO2)*	Increased interaction with nuclear factor κB	↑ Transrepression		
LRH-1	Phosphorylation	Ser238	Unknown	↑		Lee *et al*., 2006 (45)
		Ser243				
	SUMOylation	Lys224	Increased correpressor interaction	↑ Transrepression	Loss at K289 leads to increased reverse cholesterol transport and diminished development of atherosclerosis in mice	Chalkiadaki and Talianidas, 2005 (46); Stein *et al*., 2014 (47); Venteclef *et al*., 2010 (48)
		Lys289	Increased correpressor interaction	↓		
LXRs	Deacetylation	Lys432 (LXR*α*)	Ubiquitination of receptor	↑	Deficiency causes impaired lipid metabolism and decrease in plasma HDL levels in mice	Defour *et al*., 2012 (49); Li *et al*., 2007 (50)
		Lys433 (LXR*β*)				
	Phosphorylation	Ser198 (LXR*α*)	NCoR recruitment	↓ (Gene specific)	Decrease leads to reduction in hepatic fat in mice on a high-fat diet	Chen *et al.*, 2006 (51); Cho *et al.*, 2015 (52); Hwahng *et al.*, 2009 (53); Torra *et al.*, 2008 (54); Yamamoto *et al.*, 2007 (55); Wu *et al.*, 2015 (56)
		Thr290	Decreased DNA binding	↓	Induction caused reduction of circulating cortisol and glucose in rats	
		Ser291	Reduced coactivator and increased corepressor recruitment			
	SUMOylation	Lys328, 434 (LXR*α*)	Increased correpressor interaction	↑ Transrepression		Ghisletti *et al.*, 2007 (57); Huang *et al.*, 2011 (58); Pascual-García *et al.*, 2013 (59)
		Lys410, 448 (LXR*β*)				

RXRs	Acetylation	Lys125 (RXR*α*)	Increased DNA binding	↑		Zhao *et al.*, 2007 (60)
	Phosphorylation	Ser260 (RXR*α*)	Reduced heterodimerization and cofactor recruitment	↓	Receptor’s resistance to degradation is strongly liked to cell malignancy	Macoritto *et al.*, 2008 (61); Matsushima-Nishiwaki *et al.*, 1996 (62); Zimmerman *et al.*, 2006 (63); Yoshimura *et al.*, 2007 (64); Adachi *et al.*, 2002 (65); Bruck *et al*., 2005 (66)
	SUMOylation	Lys108 (RXR*α*)	Unknown	↓		Choi *et al.*, 2006 (67); Schneider *et al.*, 2013 (68)

#### FXR phosphorylation

FXR can be phosphorylated at serine 135 and serine 154 by protein kinase C (42). These residues are situated in the DNA-binding domain of the receptor (Fig. 1), and their phosphorylation increases binding to the PPAR*γ* coactivator 1*α*, leading to enhanced FXR transcriptional activity without affecting DNA binding or subcellular localization [Fig. 2(A); Table 1] (42).

Shneider *et al.* (69) also reported increased activity of FXR by phosphorylation, showing that function and nuclear localization of FXR are regulated by adenosine triphosphatase class 1 type 8B member (also known as FIC1). Mutations in this adenosine triphosphatase result in progressive familial intrahepatic cholestasis type 1 and benign recurrent intrahepatic cholestasis (70), both caused by the accumulation of hepatic bile acids ultimately resulting in liver failure. In this study, the authors argue that protein kinase C*ζ*–mediated phosphorylation of FXR is initially induced by FIC1, which, in turn, lea1ds to the enhanced transcription of FXR target genes.

Recently, adenosine monophosphate protein kinase (AMPK) was shown to directly interact and phosphorylate FXR at serine 250 (Fig. 1) (43). Activation of AMPK by metformin, a commonly used drug for the treatment of type 2 diabetes, leads to the inhibition of FXR transcriptional activity by impairing coactivator binding. Reduced FXR target gene expression by metformin-activated AMPK decreases fecal bile acid excretion in wild-type mice, and it aggravates liver injury in an animal model of intrahepatic cholestasis (43).

Overall, these results demonstrate the complexity of FXR regulation by phosphorylation, with different kinases acting on different residues and having opposite outcomes, which highlights the role of this modification on the aberrant activity of FXR and its potential role in bile acid–related diseases (Table 1).

#### FXR SUMOylation

FXR is SUMOylated by SUMO1 *in vitro* and *in vivo* on 2 residues: K122 and K275 (Fig. 1) (44). SUMOylation reduces FXR ligand–induced transactivation and recruitment to its target gene promoters without affecting its nuclear localization [Fig. 2(A)]. A recent study showed that ligand-induced SUMO2-FXR is necessary for the transrepression of several proinflammatory genes (41), a process that does not involve the direct binding of FXR to DNA [Fig. 2(A)]. Furthermore, in a mouse model of diet-induced obesity, hepatic FXR is strongly acetylated at K217. Using a mutant version of FXR mimicking acetylated K217 in lean mice (K217Q), this study demonstrated that enhanced FXR acetylation induced the expression of several hepatic proinflammatory genes, leading to increased macrophage infiltration, which was inversely correlated with the levels of sumoylated FXR [Fig. 2(B); Table 1] (41). This is partly due to decreased interaction between FXR and the SUMO-conjugating enzyme PIAS*γ*. This work not only proved how different PTMs can act in the regulation of NR activity in a coordinated manner, but it also provided evidence of the therapeutic potential of modulating PTMs, whereby targeting (inhibiting) FXR acetylation at K217 leading to the SUMOylation of the receptor could result in ameliorated hepatic inflammation and increased glucose tolerance in obese individuals, as shown in animal models of the disease.

#### FXR *O*-GlcNAcylation

FXR was recently demonstrated to be *O*-GlcNAcylated at its N-terminal activation function (AF)-1 domain via *O*-GlcNAc transferase. *O*-GlcNAcylation increases FXR expression, transcriptional activity, and stability while retaining its nuclear localization (71). It was speculated that at low glucose concentrations FXR binds to active corepressor complexes, which may be then modulated when FXR is *O*-GlcNAcylated at high glucose concentrations. However, the exact mechanism underlying the enhanced transcriptional activation remains to be elucidated.

Intriguingly, *O*-GlcNAcylation of LXR (see later) and activation of 2 other transcription factors, that is, carbohydrate-responsive element-binding protein (72) and sterol-responsive element-binding protein 1 (73), induce the expression of fatty acid synthase (FAS), whose dysregulation has been linked to the pathogenesis of metabolic diseases (74). On the contrary, *O*-GlcNAcylation of FXR has the opposite effect—reducing FAS expression. Details of this balancing act of FXR *O*-GlcNAcylation over the modification of these other regulators of FAS expression remain to be identified.

FXR not only plays an important metabolic role, but it also elicits strong anti-inflammatory properties (75, 76). Targeting the receptor’s activity with a full agonist may lead to serious side effects, such as a decrease in circulating high-density lipoprotein (HDL) levels and systemic cholesterol accumulation as a consequence of reduced bile acid synthesis (77). Indeed, a recent clinical trial assessing the efficacy of the potent FXR activator obeticholic acid for the treatment of steatohepatitis was interrupted, partly due to induced lipid abnormalities in obeticholic acid–treated patients, including increased circulating total cholesterol and low-density lipoprotein with decreased HDL (78). Therefore, selective modulation of FXR function by tissue-specific agonists or altering its posttranslational modifications could prove to be alternative effective therapeutic approaches.

### Liver receptor homolog-1

Liver receptor homolog-1 (LRH-1), also known as fetoprotein transcription factor, is an orphan NR member of the fushi tarazu factor-1 subfamily. It is highly expressed in the intestine and liver, where it plays several functions ranging from development to cholesterol and bile acid homeostasis (79). In contrast to other lipid-activated NRs, including LXR and FXR, LRH-1 binds with high affinity as a monomer to its DNA response elements to induce transcription of its target genes.

#### LRH-1 phosphorylation

LRH-1 is phosphorylated at several serine residues located in its hinge and ligand-binding domains by the mitogen-activated protein kinase extracellular signal-regulated kinases (45). These include serine 238 and serine 243 (Fig. 1), whose phosphorylation is induced by phorbol myristate acetate stimulation in both hepatic HepG2 and adenocarcinoma HeLa cells to increase the receptor’s transactivation activity (Table 1). LRH-1 phosphorylation by extracellular signal–regulated kinase may implicate a novel role for this receptor in proliferation in response to mitogenic stimuli.

#### LRH-1 SUMOylation

LRH-1 SUMOylation, by SUMO-1, was first described at lysine 224, located in the hinge region of the receptor (Fig. 1). This causes the protein to be sequestered into nuclear bodies, inhibiting its transcriptional capacity (Table 1) (46). A later study showed that this modification is also responsible for inducing LRH-1’s transrepressive activity *in vitro* and in a mouse model of hepatic acute phase response (48). The transrepression elicited upon LRH-1 SUMOylation requires the presence of the NCoR subunit G protein pathway suppressor 2, which acts as a docking site stabilizing its interaction with the NCoR1/HDAC3 corepressor complex. Additionally, in an acute phase response setting ligand-activated LXR*β* suppresses the expression of inflammatory genes, which is mediated through SUMOylation of the LRH-1 receptor (57, 80). This poses the idea of SUMOylation as a common mechanism that may regulate the crosstalk between the transrepressive activities of different NRs, as has been shown for PPAR*γ* and LXR (57, 80).

Further evidence for a pathophysiological role for LRH-1 SUMOylation has now been provided in the context of cardiovascular disease. Loss of LRH-1 SUMOylation at residue lysine 289 leads to an increased reverse cholesterol transport and reduced atherosclerosis in the low-density lipoprotein receptor knockout mouse model (Table 1) (47). This is accompanied by the increased expression of a subset of LRH-1 target genes, mainly involved in hepatic cholesterol homeostasis. This gene-selective effect on expression was explained by the reduced binding of the non-SUMOylatable LRH-1–K289R form to the prospero homeobox protein 1 corepressor. This study is highly relevant as one of the very few directly addressing the impact of changes in NR modifications on disease progression at a whole-body level.

Owing to LRH-1 effects on bile acid, cholesterol, and glucose homeostasis, this receptor has been considered a potential therapeutic target for the treatment of metabolic diseases, especially in liver, where it is highly expressed. However, how PTMs specifically regulate LRH-1 activity *in vivo* needs to be confirmed with further studies. It also remains to be investigated whether other PTMs such as acetylation play a role in finely tuning the activity of this receptor.

### LXRs

The LXR family consists of 2 different isotypes, LXR*α* and LXR*β*, with their names deriving from the initial isolation of the LXR*α* isotype from human liver (81, 82). The 2 isotypes share ∼75% sequence homology in both their DNA-binding domain and ligand-binding domain, and they differ mainly on their N-terminal sequence and their expression pattern (83). LXR*α* is predominantly expressed in liver and other metabolically active tissues and cell types, such as kidney, intestine, and macrophages, whereas LXR*β* is ubiquitously expressed. Both LXRs regulate transcription by forming permissive heterodimers with RXR (84), that is, they can be activated by ligands for each heterodimeric partner. LXRs are physiologically activated primarily by oxidized metabolites of cholesterol (84, 85), the cholesterol precursor desmosterol (86, 87), as well as a number of synthetic ligands (88, 89). These receptors play a crucial role in the regulation of cholesterol and fatty acid homeostasis (90) but also act as modulators of inflammation and immunity (91). Therefore, they are promising targets for the treatment of several pathologies with a metabolic and inflammatory component, such as atherosclerosis (92). A number of PTMs have now been reported to regulate their stability and transcriptional capacity.

#### LXR acetylation

**Figure 3.**
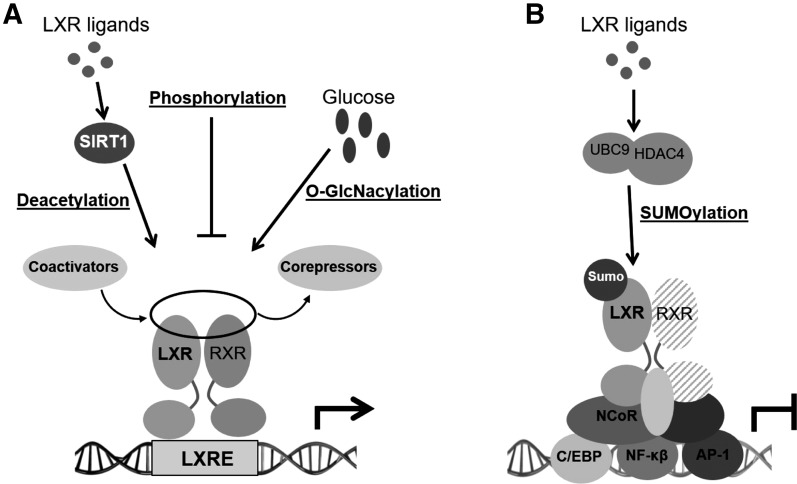
Effects of posttranslational modifications on LXR transcriptional activation (A) and transrepression (B). (A) Deacetylation by LXR agonists or *O*-GlcNAcylation by glucose induces LXR target gene expression, whereas phosphorylation has a gene-specific effect. (B) LXR transrepression of inflammatory gene expression is promoted by SUMOylation of the receptor, which consequently increases LXR avidity for the NCoR complex.

Removal of acetyl groups from lysines in LXRs by the SIRT1 deacetylase (at K432 in LXR*α* and K433 in LXR*β*, Fig. 1) promotes the receptor’s ubiquitination and subsequent degradation by the proteasome, while being a positive regulator of its transcriptional activation [Fig. 3(A); Table 1] (50). Li *et al.* (50) suggested that ligand-dependent deacetylation of LXR and consequent degradation leads to its clearance from gene promoters, which facilitates the next round of transcription and thus increases the expression of its target genes. Interestingly, this study also demonstrated that animals deficient in *Sirt1* showed higher levels of LXRA protein and displayed impaired lipid metabolism and defective reverse cholesterol transport in part due to reduced *Abca1* expression and subsequent decrease in HDL levels, as well as increased hepatic and testicular cholesterol levels (Table 1). This mechanism was further supported by a study in human skeletal muscle where SIRT1 was shown to regulate the expression of the lipogenic LXR target gene *Srebp1c* (49).

#### LXR *O*-GlcNAcylation

Albeit controversial, a study claiming that glucose is capable of activating LXRs and act as their ligand at physiological concentrations raised new insights into how LXR activity may be directly regulated by other mechanisms besides ligand binding (93). This was followed by a study on human hepatic cells and an animal model of streptozotocin-induced insulitis and diabetes in which it was reported that LXRs undergo *O*-GlcNacylation in response to glucose *in vitro* or by refeeding *in vivo* [Fig. 3(A)] (94). The authors propose that previously reported effects by glucose (93), a hydrophilic compound, were caused by its downstream signaling, presumably through posttranslational modifications, rather than the direct binding to the LXR highly hydrophobic ligand-binding domain. This study also argues that this modification affects the expression of the lipogenic transcription factor *Srebp1c*, although the exact mechanisms through which *O*-GlcNacylation regulates LXR activity need to be further elucidated.

#### LXR phosphorylation

LXR*α* is phosphorylated at serine 198 (Fig. 1) (Ser198 or Ser196 in the human and murine sequence, respectively) both *in vitro* and in atherosclerotic plaques of apolipoprotein E–deficient mice (51, 54, 56). This modulates LXR*α* transcriptional activity in a gene-selective manner [Fig. 3(A); Table 1] and is enhanced by both endogenous [24(*S*),25-epoxycholesterol] and synthetic (T0901317 and GW3965) LXR ligands (54, 56). In a murine macrophage cell line stably expressing LXR*α*, ligands for the RXR receptor such as the 9-*cis*–retinoic acid and bexarotene inhibited Ser198 phosphorylation, leading to changes in LXR/RXR-regulated gene expression, particularly on genes sensitive to changes in LXR*α* phosphorylation at this residue, such as *Ccl24* (54). The Ser198 residue is located in the hinge region of LXR*α* and was shown to be targeted by casein kinase 2 (54). Peptide molecular modeling studies suggest that Ser198 phosphorylation affects LXR*α* conformation, possibly influencing the recruitment of cofactors, such as NCoR (54, 56). Further evidence also supports these gene-selective changes by LXR*α* phosphorylation. For instance, macrophage expression of CCR7 is markedly induced by LXR*α* when the receptor is not phosphorylated at Ser198 (56). This is associated with decreased levels of chromatin repression marks (H3K9me3 and H3K27me3) at the *Ccr7* locus in cells expressing the nonphosphorylated version of the receptor.

Phosphorylation of LXR*α* by other kinases, including protein kinase A, has been reported at several residues (Ser195, Ser196, Thr290, Ser291) (Fig. 1) in rat primary hepatocytes and mouse liver, although detailed mutagenesis studies were not performed (55). Protein kinase A–mediated phosphorylation of LXR*α* leads to the repression of *Srebp1c* expression, a well-established LXR target gene, caused by decreased binding of the RXR/LXR*α* heterodimer to DNA, as well as reduced coactivator (steroid receptor coactivator-1) and increased corepressor (NCoR) occupancy (Table 1). The regulation of LXR*α* phosphorylation by cholesterol and oxysterols (54) prompted other studies investigating the effect of nutrient-regulated kinases. Oltipraz [4-methyl-5-(2-pyrazinyl)-1,2-dithiole-3-thione] is a member of the dithiolethione family, a series of compounds naturally found in cruciferous vegetables with a broad range of therapeutic uses, including chemoprevention (95) and liver fibrosis (96). Interestingly, oltipraz attenuates LXR*α* phosphorylation at an unspecified serine residue in mouse liver through the inhibition of p70 ribosomal S6 kinase-1 (53), a major downstream effector of the mammalian target of rapamycin signaling pathway. This decrease in LXR*α* serine phosphorylation leads to a reduction in *Srebp1c* target gene expression in culture. Additionally, oltipraz administration to mice fed a high-fat diet caused a decrease in hepatic fat content, pointing to oltipraz and the modulation of LXR phosphorylation as potential therapeutic targets for the treatment of fatty liver. Furthermore, a recent report examining the metabolic effects of metformin showed that this compound induces LXR*α* phosphorylation (at a threonine residue) in rat pituitary cells, which in turn causes a reduction in the expression of its target gene *Pomc*, a precursor of the adrenocorticotropic hormone, leading to an overall reduction of systemic cortisol and glucose (52). In this context, LXR*α* threonine phosphorylation is shown to be induced by activated AMPK, which had been previously associated with the pleiotropic actions of metformin.

#### LXR SUMOylation

As the mechanistic basis for the transcriptional repression of proinflammatory genes, Ghisletti *et al.* (57) initially demonstrated that ligand-induced SUMOylation of LXR is required for its interaction with the NCoR corepressor in mouse primary macrophages and RAW264.7 macrophage-like cells [Fig. 3(B); Table 1]. In addition to synthetic ligands, these authors demonstrated that SUMOylation was promoted by the LXR endogenous ligands 22(*R*)-hydroxycholesterol, 24(*S*),25-epoxycholesterol, and 24(*S*)-hydroxycholesterol. In contrast to PPAR*γ*, whose SUMOylation is dependent on PIAS1 and SUMO1 (97), LXR-mediated transrepression involves SUMOylation by SUMO2 and SUMO3, with HDAC4 acting as the SUMO E3 ubiquitin ligase. It was later shown that the interaction between SUMOylated LXR and NCoR was facilitated by Coronin 2A, a member of the actin-binding protein family that acts both as a docking site for LXR and an exchange factor for NCoR, proving necessary for the derepression of several nuclear factor κB–induced proinflammatory gene promoters (58). Moreover, a later study showed that mutant forms of both LXRs lacking SUMO acceptor sites (LXR*α* K328R/K434R and LXR*β* K410R/K448R) (Fig. 1) have a decreased capacity to prevent the binding of the proinflammatory signal transducer and activator of transcription 1 transcription factor to the *Nos2* promoter (59), further establishing the importance of LXR SUMOylation on its transrepressive capacity. However, this transrepression model has now been challenged. Ito *et al.* (98) recently postulated that repression of inflammatory genes by LXRs is dependent on changes in cellular lipid metabolism rather than SUMOylation of the receptor. In their report, the authors proposed a mechanism whereby LXR-dependent expression of the adenosine triphosphate–binding cassette transporter A1 (ABCA1), which mediates intracellular cholesterol efflux to apolipoprotein A1, is critical for the repression of proinflammatory genes by LXR. Increased ABCA1 expression leads to a decrease in membrane cholesterol levels as a result of a higher rate of cholesterol efflux by ABCA1, thus increasing membrane permeability and disrupting Toll-like receptor signaling due to the inability of Toll-like receptors to recruit its signal transducers. This study demonstrates that LXRs are capable of strong repressive actions even in the absence of SUMOylation in immortalized mouse embryonic fibroblasts *in vitro*, suggesting that NR activity is regulated by different independent pathways. It would be interesting to assess whether this SUMOylation-independent transrepression mechanism also occurs in other cell types and under physiological conditions. Thus, depending on the cell type and disease context, these 2 models may not be mutually exclusive. In any case, it still remains unclear what the consequences are of altering LXR SUMOylation on inflammatory diseases or other pathophysiological contexts.

These studies highlight the importance of phosphorylation and other PTMs on LXR activity. In the future, it will be exciting to uncover the exact mechanisms underlying these changes and the impact these have on the function of the receptor *in vivo* in the context of metabolic or inflammatory diseases.

### RXRs

The RXR family consists of 3 different NRs, encoded by 3 different genes: RXR*α*, RXR*β*, and RXR*γ* (99). These receptors respond to retinoids (100) or vitamin A derivatives, although the fact that retinoids have not been found in animal tissues indicates that RXRs may also have other endogenous ligands (101). They have the unique capacity to be able to form homodimers, as well as heterodimers, with a wide range of other NRs (102), including LXR and FXR, thus playing a role in a variety of developmental and metabolic functions.

#### RXR acetylation

Zhao *et al.* (60) were the first to show that RXR*α* and RXR*γ* are targets of the p300 acetyltransferase, which induces both cell proliferation and apoptosis in a context-dependent manner. In the case of RXR*α*, acetylation at lysine 125 (Fig. 1) was proven to increase the receptor’s transcriptional activity in culture by promoting a stronger binding of the receptor to DNA (Table 1). In their study, the authors also reported that acetylation of RXR*α* by p300 is reduced by the orphan receptor thyroid receptor 3 through competition for RXR*α* binding. Binding of RXR*α* to thyroid receptor 3 was further increased by the RXR ligand 9-cRA, which led to attenuated p300-induced cell proliferation via RXR*α*, suggestive of a crosstalk between different NRs being modulated by their PTMs.

#### RXR phosphorylation

Phosphorylation of human RXR*α* was initially identified on serine 260, located at the ligand-binding domain (Fig. 1) and shown to attenuate its transcriptional activation through heterodimerization with the vitamin D receptor in Ras-transformed keratinocytes (103). Phosphorylation at Ser260 by the mitogen-activated protein kinase led, in part, to the resistance of these cells to growth inhibition by 1,25-dihydroxyvitamin D_3_, the active form of vitamin D. Recently, the same group showed that RXR*α* phosphorylation at this residue affects the RXR*α*/vitamin D receptor heterodimer, causing impaired cofactor recruitment and subsequent reduced transactivation (Table 1) (61). Phosphorylation of RXR*α* on Ser260 is also involved with increased resistance to proteolysis (104) and loss of heterodimeric activity (64). Accumulation of this receptor was previously reported on hepatocellular carcinoma cells (62) and murine hepatic tumors (105), and it is suspected that the malignancy of these cells is caused in part by the loss of activity of phosphorylated RXR*α* and increased cell proliferation. Receptor accumulation is due to resistance to proteosomal degradation by the phosphorylated RXR*α* form (65). Consistently, RXR*α* is highly ubiquitinated in healthy human liver, whereas in human hepatocarcinoma tissues and cell lines, the receptor is hyperphosphorylated and thus resistant to degradation. This strong correlation between RXR phosphorylation and cell malignancy led to the notion of targeting RXR*α* phosphorylation as a therapy for liver (106) and colorectal cancers (107). Intriguingly, the same Ser260 residue is phosphorylated by c-jun N-terminal kinase in response to interleukin-1*β*, which leads to the rapid nuclear export and subsequent degradation of RXR*α* (63).

Several studies have demonstrated that RXR*α* activity diminishes in response to anisomycin, a stress stimulus that inhibits protein synthesis (66, 108). This modulation of RXR*α* activity is caused by the activation of the mitogen-activated protein kinase kinase-4 and its downstream kinase c-Jun N-terminal kinase, which phosphorylate RXR*α* at Tyr249 (108), as well as 3 different residues at the AF-1 domain (Ser61, Ser75, and Thr87 or Ser56, Ser70, and Thr82 in humans) and 1 residue (Ser265 or Ser260 in humans) located within the AF-2 domain (66). Notably, similar to LXR*α* phosphorylation at Ser198 (78), RXR*α* phosphorylation at Ser265 inhibits transcription of a specific subset of RXR target genes in a promoter-specific manner (66).

Overall, these studies suggest that the impact of RXR*α* phosphorylation on the receptor’s activity is dependent on cell type and experimental conditions. However, the impact of RXR*α* phosphorylation on physiology needs to be further elucidated.

#### RXR SUMOylation

In addition to the previous modifications, RXR*α* has been shown to interact with ubiquitin-conjugating enzyme 9, a SUMO-conjugating E2 enzyme that mediates the SUMOylation of the receptor (67). RXR*α* is modified by SUMO-1, with lysine 108, located in the variable amino-terminal activation domain (AF-1) region of the receptor (Fig. 1), being the main SUMO acceptor site. This SUMO modification of RXR*α* negatively regulates its transcriptional activity (Table 1) and, interestingly, can be reversed by the SUMO-specific protease SUSP1, further confirming the importance of posttranslational modifications as specific regulators of RXR*α* activity. A more recent study showed that RXR*α* is also SUMOylated in response to tumor necrosis factor-α stimulation in a human hepatocellular carcinoma cell line, suggesting an interesting crosstalk between proinflammatory stimuli and RXR activity through the induction of PTMs (68).

As RXRs form heterodimers with a range of other NRs, including PPARs, LXRs, and FXRs, they hold a unique potential to play a diverse array of roles modulating multiple metabolic systems. Likewise, posttranslational modifications of RXR could strongly alter RXR heterodimer-regulated metabolic pathways. As studies identifying PTMs in RXR and its heterodimeric partners continue to emerge, it will be interesting to explore how combinatorial modifications of these modifications affect the activity of specific heterodimers in homeostatic as well as altered metabolic states observed in disease.

## Future Perspectives

NRs are involved in a vast range of biological processes, including metabolism, immunity, development, and reproduction. Their modulatory roles are often dysregulated in a number of pathologies through several mechanisms, including changes in their PTMs. Therefore, promoting these changes as a means to ultimately modulate NR activity has begun to attract considerable attention. NRs have been considered important drug targets for decades. Classically, drug development programs have focused their efforts on the identification of specific ligands that either activate or antagonize NR signaling, sometimes in a context- or tissue-specific manner. As we learn how posttranslational modifications finely tune NR actions, and how these PTMs are altered in pathological situations, pharmacological or genetic manipulation of these modifications represents an alternative therapeutic avenue that is starting to be explored.

For example, several studies have now linked the phosphorylation status of the estrogen receptor *α* in breast tumors, with resistance to endocrine treatment and overall clinical outcomes (109). This is not restricted to steroid receptors. Recently, based on initial observations that phosphorylation of PPAR*γ* at Ser273 is linked to obesity and insulin resistance (110, 111), a drug screening effort on 780 different Food and Drug Administration–approved drugs using disruption of PPAR*γ* phosphorylation as an endpoint (rather than PPAR*γ* classical ligand activation) was reported (112). These efforts identified that imatinib mesylate (Gleevec), a well-established anticancer drug, increases insulin sensitivity and overall improves the phenotype of mice fed a high-fat diet by blocking Ser273 PPAR*γ* phosphorylation (112).

Overall, 1 of the remaining challenges that needs to be addressed is gaining a better understanding of the complex relationships between PTMs in various pathological contexts in order for the development of these alternatively targeted therapeutics to become a reality for a larger number of NRs.
